# A Novel Individual-based Determination of Postoperative Cognitive Dysfunction in Mice

**DOI:** 10.14336/AD.2019.1029

**Published:** 2020-10-01

**Authors:** Jing Zhong, Jun Li, Changhong Miao, Zhiyi Zuo

**Affiliations:** ^1^Department of Anesthesiology, University of Virginia, Charlottesville, Virginia, USA.; ^2^Department of Anesthesiology, Fudan University Shanghai Cancer Center, Shanghai, China; Department of Oncology, Shanghai Medical College, Fudan University, Shanghai, China.

**Keywords:** Individual animal-based assessment, learning and memory, mice, postoperative cognitive dysfunction

## Abstract

Postoperative cognitive dysfunction (POCD) is a significant clinical issue. Aging is a risk factor for POCD. It is known that not every patient develops POCD. This situation shall be similar in animals. Determination of POCD is individual-based in humans but group-based in animal studies. This difference prevents effective evaluation of biomarkers and interventions for POCD in preclinical studies. The objective of this study was to determine whether individual animal could be assessed for POCD by a system similar to that for patients. Seven-week old CD1 and 18-month old C57BL/6 male mice were subjected to right carotid arterial exposure under isoflurane anesthesia. Mice were evaluated by Barnes maze and fear conditioning either post-surgery alone or both prior to surgery and post-surgery. Surgery increased the time to identify the target box in Barnes maze when tested one day or 8 days after the training sessions and reduced freezing behavior in fear conditioning test. This phenomenon occurred in 7-week old animals with and without evaluation before the surgery and in 18-month old mice evaluated before and after surgery. Based on the method and criteria used for a human whose cognition was evaluated before and after surgery to assess individual decline of cognition, 7 in 21 mice in the surgical group and 1 in 21 mice in control group of 7-week old mice had cognitive dysfunction. Among 18-month old mice, 13 in 21 mice in the surgical group and 2 in 20 mice in the control group had cognitive dysfunction. The incidence of cognitive dysfunction in mice with surgery was higher than that in control mice no matter whether young adult (P = 0.045) or old mice (P < 0.001) were considered. These results indicate that surgery induces POCD in mice. Individual animal-based assessment can be used to identify animals with POCD.

Postoperative cognitive dysfunction (POCD) is a significant clinical issue that is associated with increased mortality and morbidity [1-3]. A mature and accepted method has been used to diagnose POCD in patients [1]. Based on this method, about 40% of patients at hospital discharge and 10% of patients 3 months after non-cardiac surgery meet the criteria in patients who are older than 60 years. About 30% younger or middle-aged patients have POCD at hospital discharge [1]. Obviously, diagnosis of POCD in patients is an individual-based assessment process.

Because of the significance of POCD, numerous studies have been performed to understand the risks for contribution of anesthetics to and mechanisms for POCD [1-4]. However, it has been a group-based assessment for POCD in animal studies [4-6]. Avoiding testing, learning and memory repeatedly in animals has been a common practice. Thus, most of the previous animal studies have tested animals only once after surgery to study POCD [4-8]. This method of evaluation is obviously different from that used in patients and will not identify an individual animal that develops POCD, which may prevent effective preclinical evaluation of therapeutic interventions in a way similar to clinical situation. In addition, determining individual changes may help identify biomarkers for POCD in animals. Thus, the objective of this study was to determine whether POCD evaluation could also be individual-based in mice. To achieve this study objective, we subjected young adults (7-week old) and older (18-month old) mice to carotid artery exposure surgery, a procedure that is performed in patients with carotid endarterectomy.

## MATERIALS AND METHODS

The animal protocol was approved by the Institutional Animal Care and Use Committee of the University of Virginia (Charlottesville, VA). All animal experiments were carried out in accordance with the National Institutes of Health Guide for the Care and use of Laboratory Animals (NIH publications number 80-23) revised in 2011. Animals had to be used in the study because the effects of surgery on learning and memory were studied. It is not possible to simulate this situation by *in vitro* models, such as cell cultures.

### Animal Groups

Seven-week old male CD1 mice from Charles River (Germantown, Maryland; body weights ranged 32 to 45 g) were used in two sets of experiments. In the first set of experiment, animals were randomly assigned to: 1) control group (not being exposed to surgery), or 2) surgery group (right carotid artery exposure). Thirteen mice per group were used in the study. Mice in this set of experiment were evaluated by Barnes maze and fear conditioning only after surgery.

In the second experiment, 7-week old male CD1 mice (body weights ranged 34 to 45 g) were randomly assigned to: 1) control, or 2) surgery group. Twenty one mice per group were used in the study. These mice were subjected to Barnes maze and fear conditioning before and after surgery.

In the third experiment, 18-month old male C57BL/6 mice (body weights ranged 35 to 40 g) from the National Institute on Aging, National Institutes of Health (Bethesda, Maryland) were randomly assigned to: 1) control, or 2) surgery group. There were 20 mice in the control group and 21 mice in the surgery group. These mice were subjected to Barnes maze, fear conditioning, novel object recognition and open field tests before and after surgery.

Barnes maze, fear conditioning, novel object recognition and open field tests were completed 24 h before the surgery in the second and third experiments. Mice in these experiments were again subjected to Barnes maze, fear conditioning, novel object recognition and open field tests that were started 7 days after surgery and completed 22 days after the surgery. Similar to clinical situation [1], each mouse was tested by Barnes maze and fear conditioning (7-week old mice) or by Barnes maze, fear conditioning, novel object recognition and open field tests (18-month old mice). These behavior tests were performed between 10 am to 4 pm. After the completion of all behavioral tests, mice were euthanized by deep isoflurane anesthesia.

### Surgery

The surgery was a right carotid artery exposure. As we described before [7, 8], mice were anesthetized by 1.8% isoflurane delivered by an agent-specific vaporizer and carried by gases contained 30% oxygen. During the procedure, the mouse was kept at spontaneous respiration. A 1.5 cm midline neck incision was made after the mouse was exposed to isoflurane for at least 30 min. The soft tissues over the trachea were retracted gently. One centimeter long right common carotid artery was dissected carefully free from adjacent tissues without any damage on vagus nerve. The wound was then irrigated and closed by using a surgical suture. The surgical procedure was performed under sterile conditions and lasted around 15 min. After the surgery, all animals received a subcutaneous injection of 3 mg/kg bupivacaine, an effective analgesic method for mice [9, 10]. The total duration of anesthesia was 2 h. No response to toe pinching was observed during the anesthesia. During anesthesia, rectal temperature was monitored and maintained at 37°C with the aid of servo-controlled warming blanket (TCAT-2LV, Physitemp instruments, Clifton, NJ). Mice in the control group were placed in a box for 2 h but without anesthesia or surgery. On the day of surgery, all surgery was performed in the morning.

### Barnes Maze

Seven days after surgery, the animals were subjected to Barnes maze in a way we previously described [11, 12] to test their spatial learning and memory. Animals were first placed in the middle of a circular platform with 20 equally spaced holes (SD Instruments, San Diego, CA). One of these holes was connected to a dark chamber called the target box. Aversive noise (85 dB) and bright light (200 W) shed on the platform were used to encourage mice to find the target box. They had a spatial acquisition phase that lasted for 4 days with 3 min per trial, 4 trials per day and 15 min between each trial. Animals then went through the reference memory phase to test the short-term retention on day 5 and long-term retention on day 12. No test or handling was performed from day 5 to day 12. The latency to find the target box during each trial was recorded with the assistance of ANY-Maze video tracking system (SD Instruments).

### Fear Conditioning

One day after the completion of Barnes maze test, mice were subjected to fear conditioning test as we previously described [11, 12]. Each mouse was placed into a test chamber wiped with 70% alcohol and exposed to 3 tone-foot shock pairings (tone: 2000 Hz, 85 dB, 30 s; foot shock: 1 mA, 2 s) with an intertrial interval of 1 min in a relatively dark room. The mouse was removed from this test chamber 30 s after the conditioning stimuli. The animal was placed back in the same chamber without the tone and shock 24 h later for 8 min. The animal was placed 2 h later into another test chamber that had di?erent context and smell from the first test chamber in a relatively light room. This second chamber was wiped with 1% acetic acid. Freezing was recorded for 3 min without the tone stimulus. The tone was then turned on for 3 cycles, each cycle for 30 s followed by 1-min inter-cycle interval (4.5 min in total). Animal behavior in these two chambers was video recorded. The freezing behavior in the 8 min in the first chamber (context-related) and 4.5 min in the second chamber (tone-related) was scored in an 8 s interval by an observer who was blind to the group assignment.

### Open field test

Eighteen-month old male C57BL/6 mice were subjected to open field study as we described before [13]. Animals were placed in the open field box for 10 min. They were placed in the box again 7 days after the surgery or at the corresponding time in the control group. This test was administered before the Barnes maze test. The time mice spent in the corner, border and center areas and the travel distance were recorded and analyzed by the ANY-maze tracking software.

### Novel object recognition test

Mice were subjected to a novel object recognition test on the day after open field test. As described before [13], two identical objects were placed in opposite sides of the objective zone in a box on the training day. A mouse was placed in the center of the box and allowed to explore it for 5 min. An animal was eliminated from the test if the total exploration time on two objects was less than 5 s. Twenty-four hours later, a novel object and a familiar object were placed in the same locations as in the training phase. The mouse was put in the center of the box and allowed to explore for 5 min. The time of exploring novel and familiar objects was recorded and analyzed by ANY-maze tracking software. The ratio of time spent with the novel object to the time on both objects was calculated.

### Statistical Analyses

All data except for the novel object recognition data of mice that explored the two objects for less than 5 s were included in the analysis. Thus, no definition of outliers was established to exclude outlier data from the analysis. Parametric results in normal distribution are presented as mean ± S.D. (n ≥ 13, n refers to the number of mice) in the figures. Non-normally distributed data are presented in box plot showing the median and 95th percentile of the data. The data from the training sessions of Barnes maze test within the same group or the body weights of different times in the same group were tested by one-way repeated measures analysis of variance followed by the Tukey test. The data from the training sessions of Barnes maze test between groups or the body weights of different times between groups were tested by two-way repeated measures analysis of variance followed by the Tukey test. The comparison of proportion of mice with cognitive dysfunction between the control group and the surgery group was performed by Fisher Exact. All other data were analyzed by an independent t test if the data were normally distributed or by rank sum test if the data were not normally distributed. A di?erence was considered significant at P < 0.05 based on two-tailed hypothesis testing. All statistical analyses were performed with SigmaPlot 14.0.

We used the same method as for human study to calculate the Z score for the diagnosis of POCD [1]. Briefly, we subtracted the postoperative test results from baseline (preoperative) data and divided the result by the corresponding S.D. generated from control group to obtain a Z score for each individual test. The sign is adjusted so that positive Z scores indicate deterioration from the baseline test [1, 14]. We treated the short retention and long retention in the Barnes maze and context-related and tone-related freezing behavior in the fear conditioning as separate tests. The composite Z scores from these four tests were calculated by summing the Z score from the individual test and then divided it by the S.D. generated from summed Z score of control group. A mouse was classified as exhibiting POCD if the Z score was 1.96 or greater in two individual tests or if the composite Z score was 1.96 or greater. This technique identified mice with POCD by comparing the changes in the test scores of an individual mouse undergoing surgery with changes in the test scores of the control group over the same time interval.

The primary outcome of this study was the proportion of mice met the criteria for cognitive dysfunction. Secondary outcomes were learning and memory decline after surgery assessed by a group-based method. We did not perform statistical power calculation before the study. The sample size of each experiment was decided based on our previous experience in this line of research [7, 13].

**Figure 1.**
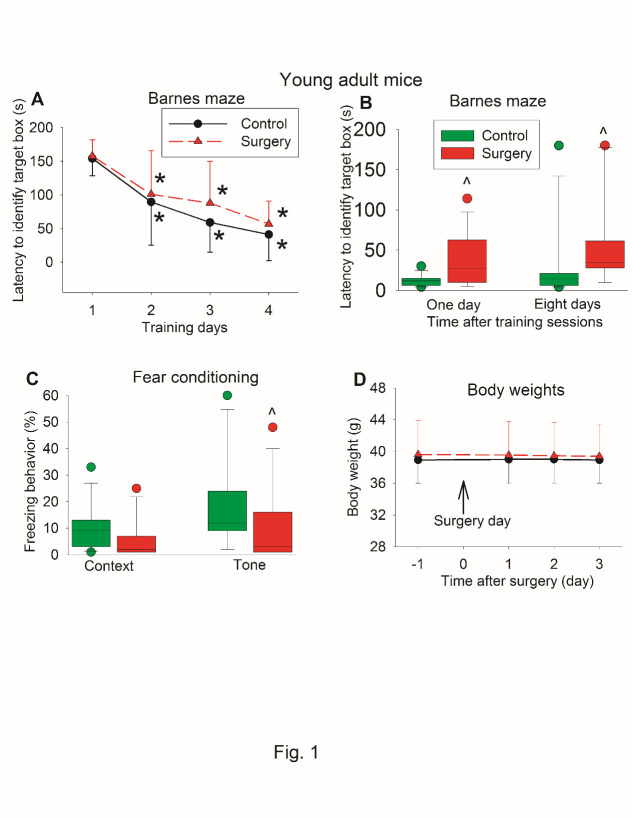
Effects of surgery on learning and memory assessed only after surgery in young adult mice. Seven-week old CD1 mice were subjected to right carotid artery exposure under isoflurane anesthesia. Barnes maze training sessions started 1 week after the surgery. (A) Barnes maze training sessions. Results are mean ± S.D. (n = 13). * P < 0.05 compared with the corresponding data on day 1. (B) Barnes maze memory phase. (C) Fear conditioning results. Results in B and C are in box plot format (n = 13). ? : lowest or highest score (the score will not show up if it falls in the 95th percentile); between lines: 95th percentile of the data; inside boxes: 25th to 75th percentile including the median of the data. ^ P < 0.05 compared with the control group. (D) Body weights. Results are mean ± S.D. (n = 13).

## RESULTS

Data from all mice in the three sets of experiments (n = 13 per group in experiment 1, n = 21 per group in experiment 2, n = 20 for the control group and = 21 for the surgery group in experiment 3) were included in analysis.

The time for young adult mice to identify the target box was decreased with training no matter whether the mice had surgery or not. Mice needed less time on day 2 to day 4 during the training sessions to identify the target box than they did on day 1 (Fig. 1A). Surgery was not a significant factor to affect the time to identify the target box during the training sessions [F(1,24) = 0.928, P = 0.345]. However, mice with surgery took longer to identify the target box one day or eight days after the training sessions (Fig. 1B). Mice with surgery also had less tone-related freezing behavior than control mice in the fear conditioning test (Fig. 1C). As an indicator for general well-being, we measured body weight. Surgery did not affect the body weight of mice during the 3 days after the surgery [F(1,24) = 0.145, P = 0.706] (Fig. 1D), suggesting that these mice with surgery did not have significant pain because body weight can be used as an indicator for pain after surgery [15].

**Figure 2.**
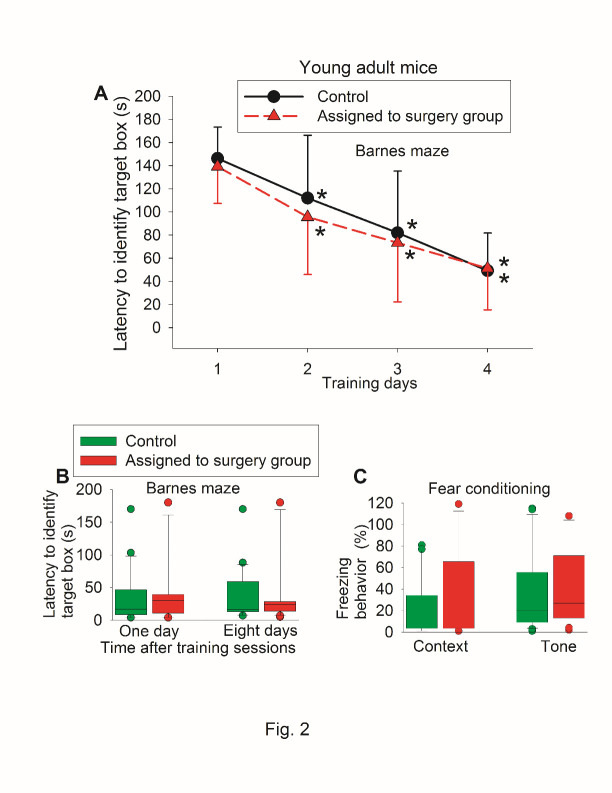
Preoperative evaluation of learning and memory of young adult mice in a self-comparison study. Seven-week old CD1 mice were used in the test. (A) Barnes maze training sessions. Results are mean ± S.D. (n = 21). * P < 0.05 compared with the corresponding data on day 1. (B) Barnes maze memory phase. (C) Fear conditioning results. Results in B and C are in box plot format (n = 21). ?: lowest or highest score (the score will not show up if it falls in the 95th percentile); between lines: 95th percentile of the data; inside boxes: 25th to 75th percentile including the median of the data.

**Figure 3.**
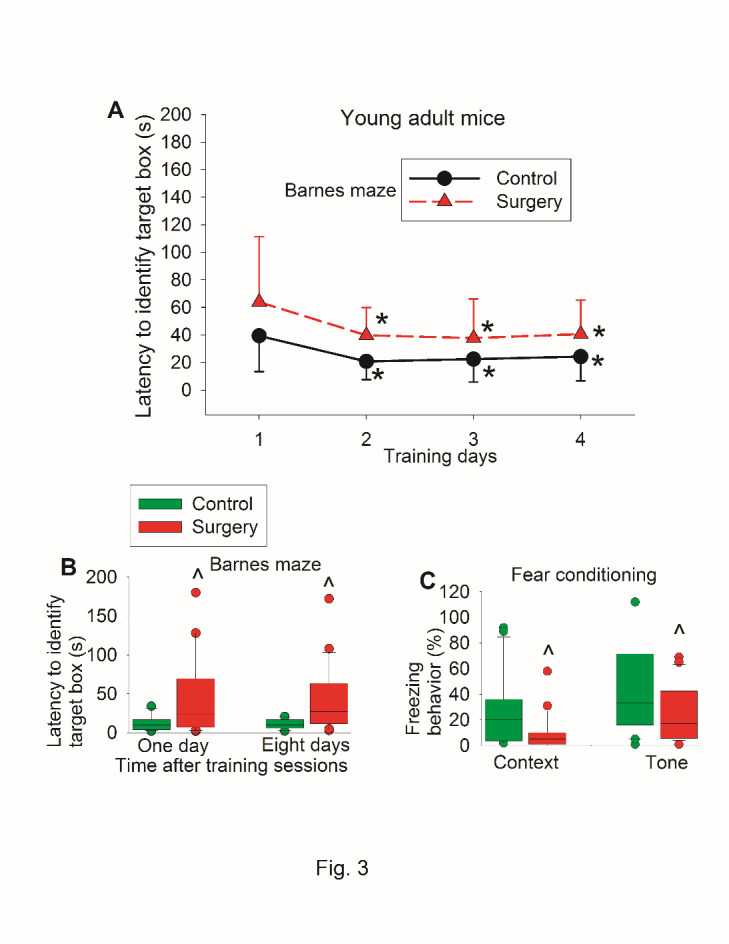
Postoperative evaluation of learning and memory of young adult mice in self-comparison study. Seven-week old CD1 mice were subjected to right carotid artery exposure under isoflurane anesthesia. Barnes maze training sessions started 1 week after the surgery that was performed 24 h after preoperative evaluation of their learning and memory (preoperative results are shown in figure 2). (A) Barnes maze training sessions. Results are mean ± S.D. (n = 21). * P < 0.05 compared with the corresponding data on day 1. (B) Barnes maze memory phase. (C) Fear conditioning results. Results in B and C are in box plot format (n = 21). ?: lowest or highest score (the score will not show up if it falls in the 95th percentile); between lines: 95th percentile of the data; inside boxes: 25th to 75th percentile including the median of the data. ^ P < 0.05 compared with the control group.

There was no difference in the performance of Barnes maze and fear conditioning between control mice and mice assigned to surgery when they were tested before the surgery (Fig. 2). When they were tested again after the surgery, control mice and mice with surgery had decreased time needed to identify the target box with increased training sessions (Fig. 3A). Surgery was a significant factor to affect the time to identify the target box during the training sessions [F(1,40) = 8.712, P = 0.005]. Mice with surgery needed more time to identify the target box one day or eight days after the surgery (Fig. 3B). Mice with surgery also had less freezing behavior in the context-related or tone-related fear conditioning test (Fig. 3C).

**Figure 4.**
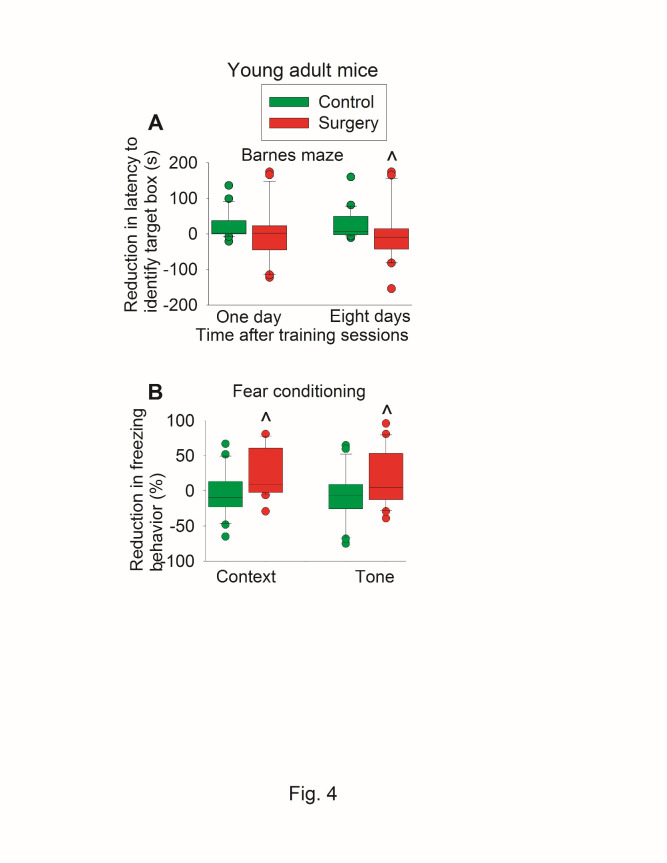
Effects of surgery on learning and memory assessed in a self-comparison fashion in young adult mice. Seven-week old CD1 mice were subjected to preoperative evaluation and postoperative evaluation of their learning and memory. Those results are shown in figures 3 and 4. The difference between preoperative values and postoperative values was calculated and presented here. (A) Barnes maze memory phase. (B) Fear conditioning results. Results are in box plot format (n = 21). ?: lowest or highest score (the score will not show up if it falls in the 95th percentile); between lines: 95th percentile of the data; inside boxes: 25th to 75th percentile including the median of the data. ^ P < 0.05 compared with the control group.

When reduction in time to identify the target box was calculated (time needed before the surgery - time after the surgery), the reduction in time for young adult mice to identify target box 8 days after the training sessions of Barnes maze test in control group was 7 (-2 to 48) s [median (25% to 75%)] vs. -9 (-41 to 12) s in surgery group (P = 0.011 for comparison) (Fig. 4A). Similarly, mice with surgery had less context-related and tone-related fear conditioning than control mice when the reduction of freezing behavior was calculated (Fig. 4B).

**Figure 5.**
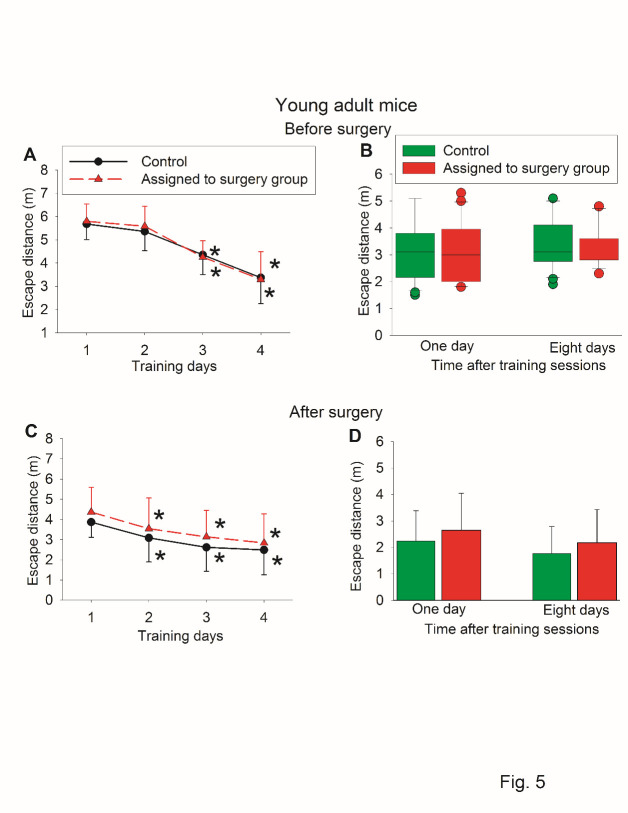
Escape distance traveled by young adult mice in Barnes maze test in self-comparison study. Seven-week old CD1 mice were subjected to right carotid artery exposure under isoflurane anesthesia. Barnes maze tests were performed before and after surgery (those results of latency to identify the target box are shown in figures 2 and 3). (A) Barnes maze training sessions before surgery. (B) Barnes maze memory phase before surgery. (C) Barnes maze training sessions after surgery. (D) Barnes maze memory phase after surgery. Results are mean ± S.D. for panels A, C and D (n = 21). * P < 0.05 compared with the corresponding data on day 1. Results in panel B are in box plot format (n = 21). ? : lowest or highest score (the score will not show up if it falls in the 95th percentile); between lines: 95th percentile of the data; inside boxes: 25th to 75th percentile including the median of the data.

Similar to the results of latency to identify the target box, the travel distance to identify target box was decreased with the training sessions. There was no difference in the travel distance one day and 8 days after the training sessions between control mice and mice assigned to a surgery group when Barnes maze test was administered before the surgery. There was also no difference in the travel distance one day (P = 0.300) and 8 days (P = 0.247) after the training sessions between control mice and mice with surgery when Barnes maze test was administered after the surgery (Fig. 5). These results suggest that travel distance to identify the target box may be a less sensitive indicator for learning and memory dysfunction than latency to identify the target box.

For the experiment using 7-week old mice, there were 6 mice in the surgery group and no mice in the control group that had a Z score more than 1.96 in two tests from the possible four tests (one or eight days after the training sessions in Barnes maze test and context-related and tone-related fear conditioning). One additional mouse from the surgery group and one mouse from the control group had a composite Z score more than 1.96. Thus, there are 7 mice in the surgery group and one mouse in the control group that met the criteria for cognitive decline (Table 1). There was a significant difference in the number of mice with cognitive decline between the two groups (P = 0.045).

**Figure 6.**
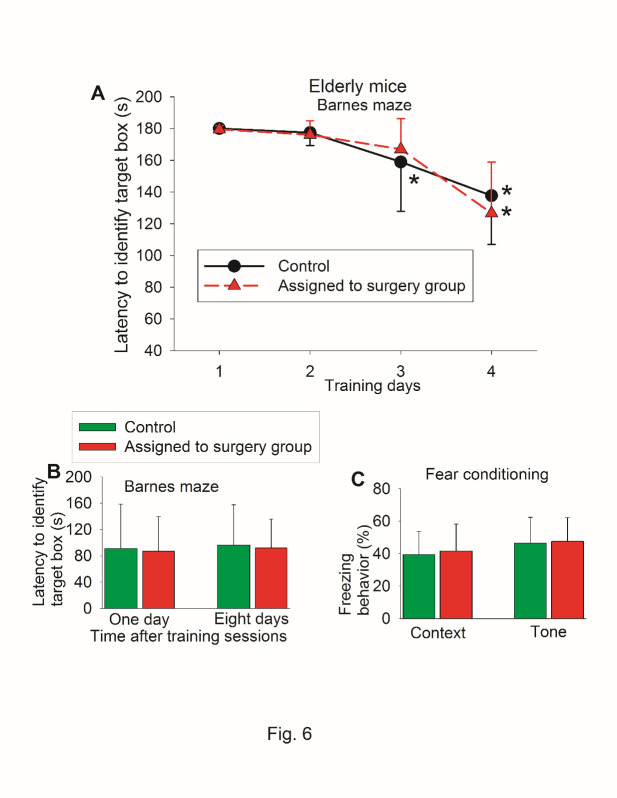
Preoperative evaluation of learning and memory of old mice in self-comparison study. Eighteen-month old C57BL/6 mice were used in the test. (A) Barnes maze training sessions. * P < 0.05 compared with the corresponding data on day 1. (B) Barnes maze memory phase. (C) Fear conditioning results. Results in all three panels are mean ± S.D. (n = 20 for control group and = 21 for mice assigned to surgery group).

**Figure 7.**
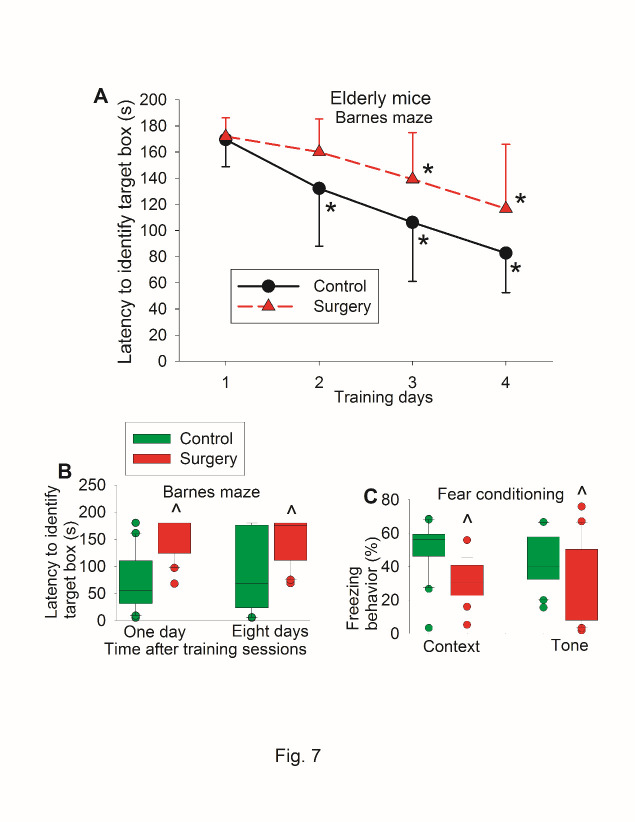
Postoperative evaluation of learning and memory of old mice in self-comparison study. Eighteen-month old C57BL/6 mice were subjected to right carotid artery exposure under isoflurane anesthesia. Barnes maze training sessions started 1 week after the surgery that was performed 24 h after preoperative evaluation of their learning and memory (preoperative results are shown in figure 6). (A) Barnes maze training sessions. Results are mean ± S.D. (n = 20 for control group and = 21 for surgery group). * P < 0.05 compared with the corresponding data on day 1. (B) Barnes maze memory phase. (C) Fear conditioning results. Results in B and C are in box plot format (n = 20 for control group and = 21 for surgery group). ?: lowest or highest score (the score will not show up if it falls in the 95th percentile); between lines: 95th percentile of the data; inside boxes: 25th to 75th percentile including the median of the data. ^ P < 0.05 compared with the control group.

To determine whether the findings in the young adult mice were reproducible and since age is a risk factor for POCD [1], we repeated the study in 18-month old mice. There was no difference in the performance of Barnes maze test and fear conditioning between control mice and mice assigned to surgery before they had surgery (Fig. 6). When they were re-tested again, surgery was a significant factor to affect the time to identify the target box during the training sessions [F(1,39) = 8.202, P = 0.007] (Fig. 7A). Mice with surgery needed more time to identify the target box one day or eight days after the surgery (Fig. 7B). Mice with surgery also had less freezing behavior in the context-related or tone-related fear conditioning test (Fig. 7C).

**Figure 8.**
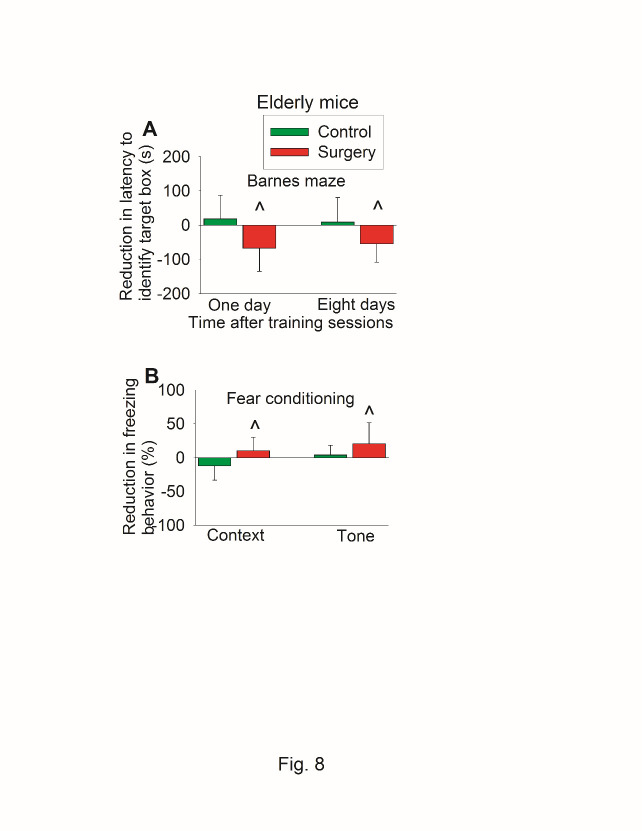
Effects of surgery on learning and memory assessed in a self-comparison fashion in old mice. Eighteen-month old C57BL/6 mice were subjected to preoperative evaluation and postoperative evaluation of their learning and memory. Those results are shown in figures 7 and 8. The difference between preoperative values and postoperative values was calculated and presented here. (A) Barnes maze memory phase. (B) Fear conditioning results. Results are mean ± S.D. (n = 20 for control group and = 21 for surgery group). ^ P < 0.05 compared with the control group.

When reduction in time to identify the target box was calculated (time needed before the surgery - time after the surgery), the reduction in time to identify target box one day after the training sessions of Barnes maze test in control group was 19 ± 69 s (mean ± S.D.) vs. -67 ± 68 s in surgery group (P < 0.001 for comparison) (Fig. 8A). Similarly, mice with surgery had less context-related and tone-related fear conditioning than control mice when the reduction of freezing behavior was calculated (Fig. 8B).

**Figure 9.**
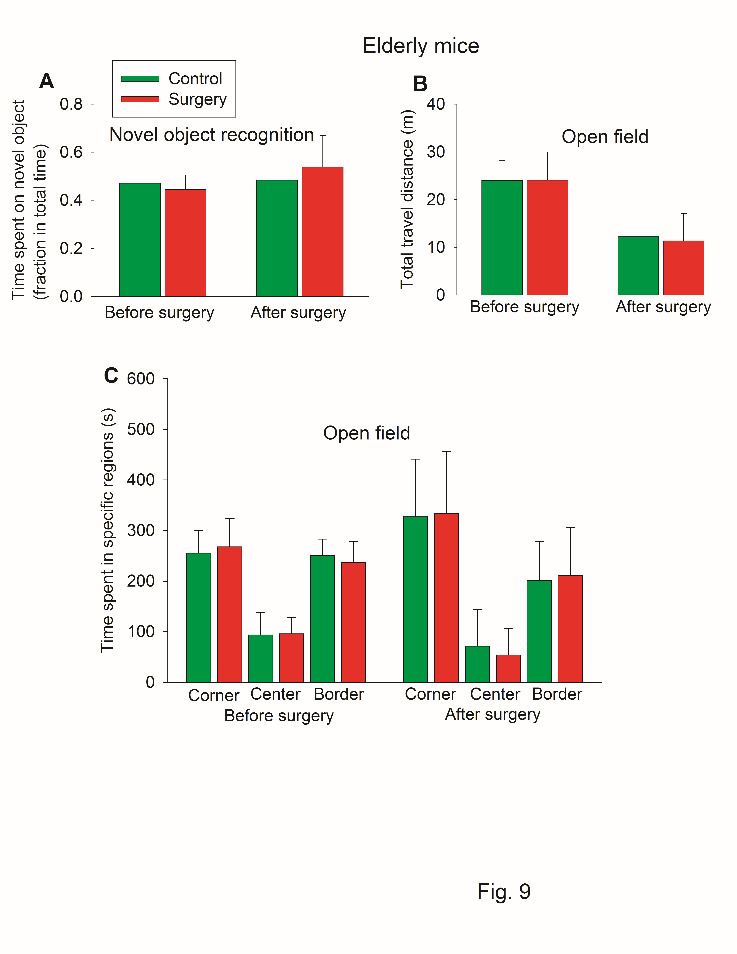
Performance of old mice in novel object recognition and open field tests in self-comparison study. Eighteen-month old C57BL/6 mice were used in the test. (A) Novel object recognition. (B) Travel distance in open field. (C) Time spent in specific regions in open field. Results in all three panels are mean ± S.D. (n = 20 for the control group and = 21 for the surgery group).

There was no difference in the time spent with the novel object between control mice and surgery mice no matter whether the test was performed before or after surgery. Of note, the ratio of time spent with the novel object to the total time with the novel object and the familiar object was around 0.5 for the control and surgery mice (Fig. 9A), suggesting that there is no preference for the novel object in these old mice. Thus, novel object recognition test may not be a good test to identify POCD in these old mice. Thus, the results of novel object recognition were not used to calculate Z scores for the determination of POCD. Of note, control mice and surgery mice traveled similar distances in open field tests (Fig. 9B). They also spent similar portions of time in the corner, center and border areas (Fig. 9C). These results suggest that the exploratory behavior and anxiety level may be similar between the control and the surgery group. Thus, the difference in the learning and memory function identified in the Barnes maze and fear conditioning may not be due to the change in the exploratory behavior and anxiety levels.

For the experiment using 18-month old mice, there were 7 mice in the surgery group and 2 mice in the control group that had a Z score more than 1.96 in two tests from the possible four tests (one or eight days after the training sessions in Barnes maze test and context-related and tone-related fear conditioning). Six additional mice from the surgery group and no additional mice from the control group had a composite Z score more than 1.96. Thus, there were 13 mice in the surgery group and 2 mice in the control group that met the criteria for cognitive decline (Table 1). There was a significant difference in the number of mice with cognitive decline between the two groups (P < 0.001).

## DISCUSSION

Consistent with previous studies [4, 7], group-based assessment showed that surgery induced learning and memory impairment because mice in the surgical group took longer than control mice to find the target box one day and eight days after the training sessions in the Barnes maze and had less freezing behavior than control mice in the fear conditioning test. However, this method will not identify individuals that develop POCD. Since not every patient develops POCD [1] and the evaluation of patients for POCD is individual-based, it is important to determine whether evaluation of animals for POCD can be individual animal-based.

We designed a system that was similar to that used in patients to evaluate POCD. This system includes evaluation of mouse learning and memory before and after surgery so that individual decline in learning and memory can be calculated. Of note, the performance of mice that were assigned to the surgical group in the Barnes maze and fear conditioning was similar to that of control mice when the tests were performed before the surgery in experiments using young adult and old mice. These results suggest that the baseline condition of these two groups was identical. However, more mice in the surgical group developed learning and memory decline than mice in the control group when they were tested after surgery and criteria used for humans to diagnose POCD [1] were used here. These results suggest that individual animal-based evaluation can be performed. Of note, the practice effects in mice are obvious because the performance of mice when tested again by Barnes maze was better than that when they were tested initially by Barnes maze test. For example, the average time to identify the target box on first training day in the first set of the Barnes maze test was around 160 s and this time in the second set of Barnes maze test was about 40 s in the control group of young adult mice. However, it is necessary to test the learning and memory function before and after surgery for each mouse to know which mouse/mice have decreased learning and memory after surgery. Nevertheless, the practice effect from repeat testing did not prevent the identification of an individual mouse with learning and memory decline and drawing a conclusion that surgery induces POCD even when they had individual-based assessment.

Group-based evaluation has been used in previous animal studies for POCD [4-8]. Our findings in this study provide initial evidence that individual animal-based evaluation is possible in both young adult and older mice. This evaluation identifies an individual animal with POCD so that the effectiveness of therapeutic interventions can be assessed in a way similar to the clinical situation. In addition, biomarker identification for POCD can then be performed in animals because the usefulness of the biomarkers can be evaluated in those animals that develop POCD. This method shall be much more powerful than the group-based assessment because many animals may not develop POCD and individual animal-based analysis shall reduce the dilution effect from those animals that do not develop POCD.

**Table 1:** Composite Z scores.

Individual mouse	Young adult mice		Elderly mice	
	Control group	Surgery group	Control group	Surgery group
1	0.926	3.725	0.131	-3.768^
2	1.828	-5.265^*	-1.232*	-0.787
3	0.074	-0.605	-0.359	-2.004^
4	0.015	-0.803	0.243	-2.093^
5	-0.596	1.700	-0.884	-4.131^*
6	0.390	-0.337	-0.293	-2.034^
7	0.800	1.051	0.221	-3.113^*
8	-2.067^	-7.026^*	-0.535	-2.933^
9	0.226	-0.641	1.506	-0.180
10	-0.334	-1.240	0.782	-2.388^*
11	1.775	-0.842	0.045	-0.542
12	-1.333	-6.122^*	0.759	-2.061^
13	-0.659	1.651	-2.619^*	0.243
14	1.334	-4.273^*	0.526	-3.510^*
15	-0.811	-0.015	1.305	-3.017^*
16	0.408	-0.406	0.576	-2.479^*
17	-0.482	-4.268^*	-1.283	-2.832^*
18	0.662	-2.619^	0.810	-1.467
19	-0.844	-0.980	-0.590	-1.064
20	-0.695	-0.876	0.892	-0.212
21	-0.617	-4.168^*		-0.921

Different mice were used for different groups. No mice were used in two study groups. The number assigned to each mouse in a group was randomly performed and were not performed in a paired format between control and surgery groups. Negative Z scores refer to decreased learning and memory after surgery (surgery group) or in the second set of tests compared to the first set of tests (control group). Positive Z scores refer to improved learning and memory after surgery (surgery group) or in the second set of tests compared to the first set of tests (control group). ^ indicates a mouse with cognitive decline as identified by composite Z score.

* indicates a mouse with cognitive decline as identified by Z scores of at least two individual tests.

We performed right carotid artery exposure, which is a necessary surgical procedure for carotid endarterectomy. We did not clamp the vessel and paid special attention not to damage the vagus nerve. This design is to simulate clinical surgical stimulation but avoid inducing brain ischemia. This procedure shall not affect extremities whose normal function is needed during the tests of learning and memory. One week was elapsed between the surgery and the beginning of learning and memory tests to avoid the in?uence of pain in these tests.

About 33% of young adult mice and 62% of older mice developed POCD in our study. This rate is similar to that reported in humans of three different ages (young, middle age and old) at hospital discharge (about 7 days after surgery) [1]. Considering that a mouse’s life-span is much shorter than a human’s life-span, the tests of Barnes maze and fear conditioning took 2 weeks to complete and most of the tests used to calculate the Z scores were performed at around 3 weeks after the surgery, the POCD identified in our study shall correspond to the delayed phase of POCD in human (occurred months after surgery). The rate of delayed POCD in human is much lower than the rates found in mice [1]. However, a precise match of the time scale between humans and mice may be difficult. In addition, the rate of delayed POCD diagnosed with the standard criteria has not been reported in humans with carotid endarterectomy.

We used two mouse strains, CD-1 and C57BL/6, in the study. Our results showed that individual-based assessment was effective in identifying mice with cognitive dysfunction in both strains, suggesting that the effectiveness of our method is not strain-dependent.

Our study has limitations. To avoid potential influence of sex hormone cycling in the learning and memory tests, we used male mice in the study. It is not known whether individual mouse-based assessment can be used in female mice for POCD. Also, since our focus was to determine whether individual mouse-based assessment of learning and memory can be performed, we did not determine the degree of neuroinflammation in our mice. Neuroinflammation is considered a critical process for POCD and our previous studies have shown that neuroinflammation has developed in this surgical model [4, 5, 7]. It would be very useful to determine whether those mice that develop POCD have worsened neuroinflammation. However, it is not possible by biochemical methods to evaluate the degree of neuroinflammation and the decline of learning and memory on the same mice because neuroinflammation disappeared a few days after surgery [5] and it is not possible to harvest brain tissues before the surgery for the comparison between the levels of inflammatory cytokines before and after surgery in the same mice. Future studies using specific markers for neuroinflammation to label brain tissues *in vivo* may make the study possible. For example, using translocator protein tracer [^11^C]PBR28 in positron emission tomography to indicate glial activation in the brain under *in vivo* conditions may be performed to suggest the degree of neuroinflammation in an individual animal [16].

In conclusion, we have shown that individual animal-based assessment for POCD can be performed in mice. This method of assessment may help evaluate the effectiveness of therapeutic interventions in preclinical studies in a way similar to clinical settings. In addition, this method of assessment shall empower the studies to identify biomarkers for POCD in preclinical studies.
